# Stereoselective Self-Assembly of DNA Binding Helicates Directed by the Viral β-Annulus Trimeric Peptide Motif

**DOI:** 10.1021/acs.bioconjchem.1c00312

**Published:** 2021-07-28

**Authors:** Jacobo Gómez-González, David Bouzada, Lidia A. Pérez-Márquez, Giuseppe Sciortino, Jean-Didier Maréchal, Miguel Vázquez López, M. Eugenio Vázquez

**Affiliations:** † Centro Singular de Investigación en Química Biolóxica e Materiais Moleculares (CiQUS), Departamento de Química Inorgánica, Universidade de Santiago de Compostela, 15782 Santiago de Compostela, Spain; ‡ Centro Singular de Investigación en Química Biolóxica e Materiais Moleculares (CiQUS), Departamento de Química Orgánica, Universidade de Santiago de Compostela, 15782 Santiago de Compostela, Spain; § Insilichem, Departament de Química, Universitat Autònoma de Barcelona, 08193 Cerdanyola, Spain

## Abstract

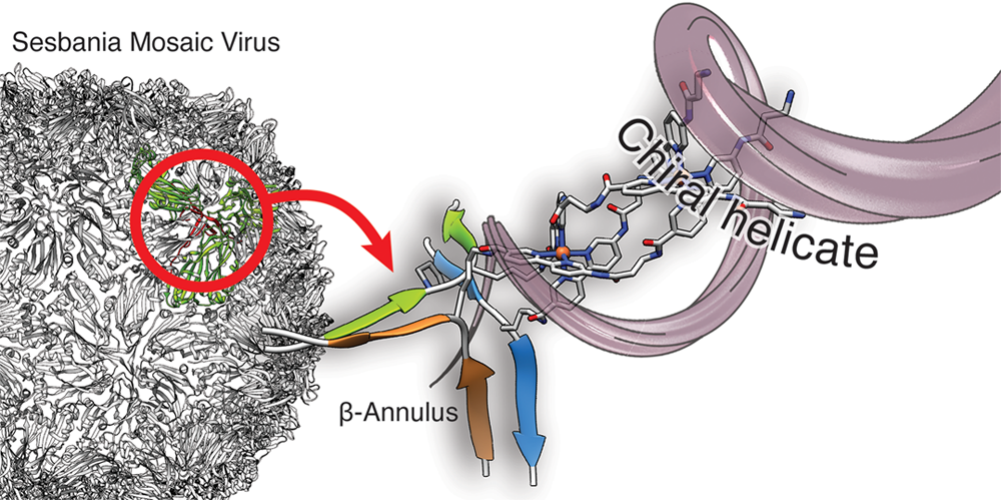

Combining coordination chemistry and peptide engineering offers extraordinary opportunities for developing novel molecular (supra)structures. Here, we demonstrate that the β-annulus motif is capable of directing the stereoselective assembly of designed peptides containing 2,2′-bipyridine ligands into parallel three-stranded chiral peptide helicates, and that these helicates selectively bind with high affinity to three-way DNA junctions.

Peptides are ideal platforms for the programmed assembly of supramolecular structures, as they encode in their sequences precise structural and functional information in their sequence. Several peptide motifs, such as coiled-coils, β-hairpins, or amphiphilic peptides, have been studied as the basis for biofunctional devices and materials.^1−9^ However, despite the enormous potential for the control of stereochemistry, nuclearity, and stoichiometry, the assembly of metal complexes driven by peptide motifs has only started to take off,^10−12^ and most examples in the literature are restricted to systems based on coiled-coils.^13−19^ As a test case to show the potential of peptide motifs to direct the assembly of metal complexes, we focused our attention on helicates,^20^ which are discrete metal complexes in which one or more organic ligands coordinate two or more metal ions,^21−23^ because of their interest in supramolecular chemistry and exciting biological applications.^24−30^ Helicates are inherently chiral species that can show right-handed (designated as *P*) or left-handed (*M*) helicity, according to the orientation in which the ligands coil around the axis defined by the metal centers. Indeed, one of the biggest challenges for the synthesis of helicates is their stereoselective assembly with controlled supramolecular chirality.^31−34^ In this context, we wanted to test whether a small trimeric peptide motif could effectively control the self-assembly of three-stranded peptide helicates, selecting a particular orientation of the ligand chains and helical chirality. In contrast to our previous approach to helicate synthesis relying on the folding of a single peptide chain,^35,36^ here we would rely on the assembly of three independent peptide monomers to template the formation of the desired helicate.

As an alternative platform to the omnipresent coiled-coils, we focused our attention on the *C*_3_-symmetric β-annulus motif, a short dodecapeptide, G^48^ISMAPSAQGAM^59^, from the *N*-terminus of the C subunits of the coat protein of the Sesbania Mosaic Virus (SeMV) capsid.^37^ Structurally, the β-annulus is a three-way junction of two-stranded β-sheets formed between residues 48–52 of each strand with residues 55–58 of the symmetric peptide chain.^38^ The backbone of the polypeptide displays a 120° turn that allows this arrangement thanks to a central residue Pro^53^ (Figure 1a,b). The β-annulus has been previously used for the formation of nanospheres and large aggregates,^39^ but its application to encode the assembly of discrete supramolecules has not yet been explored. Upon inspection of the β-annulus structure (PDB code 1X33),^40^ we realized that the three symmetrically equivalent Ser^54^ residues located at the center of the β-annulus were ideally positioned to serve as anchor points for the introduction of the helicate strands composed by two 2,2′-bipyridine building blocks (βAlaBpy) in tandem (Figure 1c and Scheme 1).^41^ Exploratory molecular modeling studies confirmed that a mutated peptide featuring a set of two chelating βAlaBpy units attached to the side-chains of mutated Lys residues at that position could coordinate a pair of metal ions forming a dinuclear helicate without significantly distorting the β-annulus structure (Figure 1d,e).

**Figure 1:**
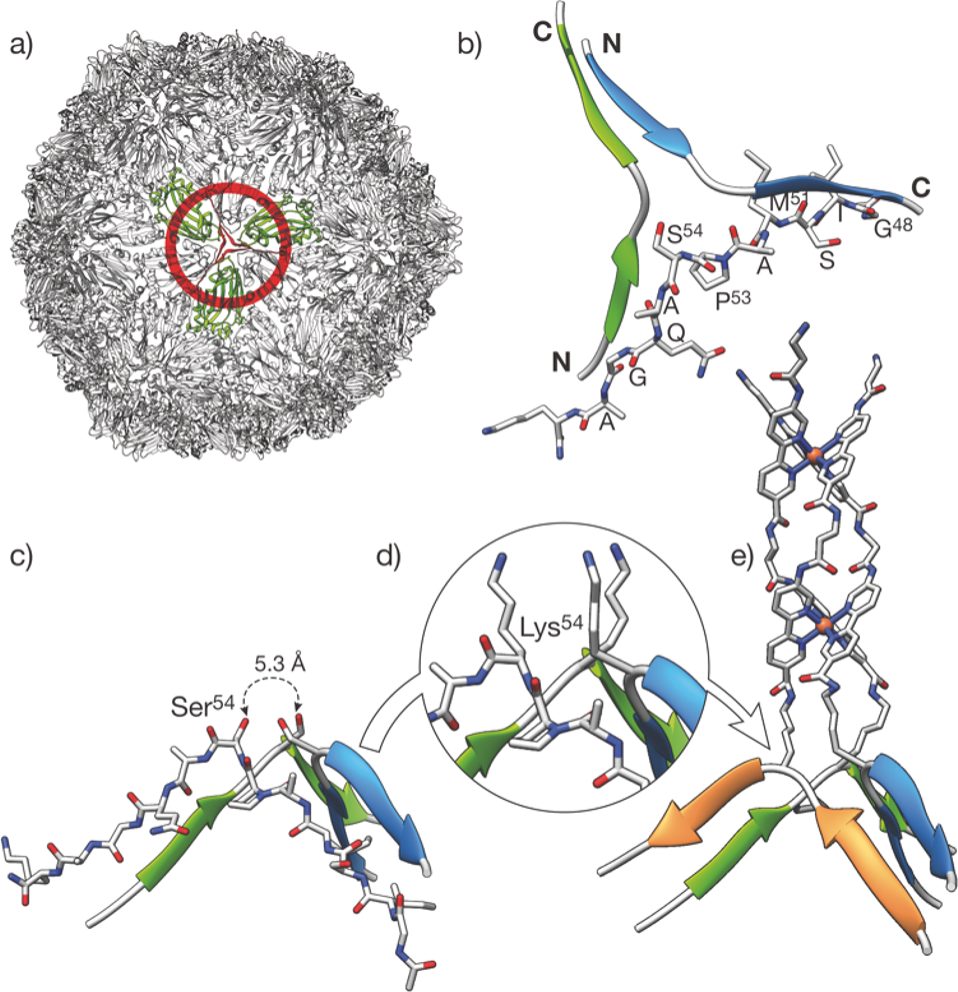
Design of the β-annulus helicate. (a) Structure of the Sesbania Mosaic Virus (SeMV) capsid, highlighting the β-annulus motif (in red), at the center of the C subunit trimer in green (PDB ID 1X33). (b) Isolated β-annulus showing the relative orientation of the three peptide chains and the natural residues in one of the symmetric peptide strands. Note the position of Ser^54^ near the center of the annulus. Bottom: modification of the β-annulus to introduce the coordinating βAlaBpy residues. (c) Detail of the β-annulus highlighting the distance between Ser^54^ residues. (d) Ser^54^ are mutated into Lys residues, that serve as anchor points for the introduction of the chelating βAlaBpy residues to yield the **β-annK(Bpy)**_**2**_ ligand precursor of the helicate shown in (e).

**Scheme 1:**
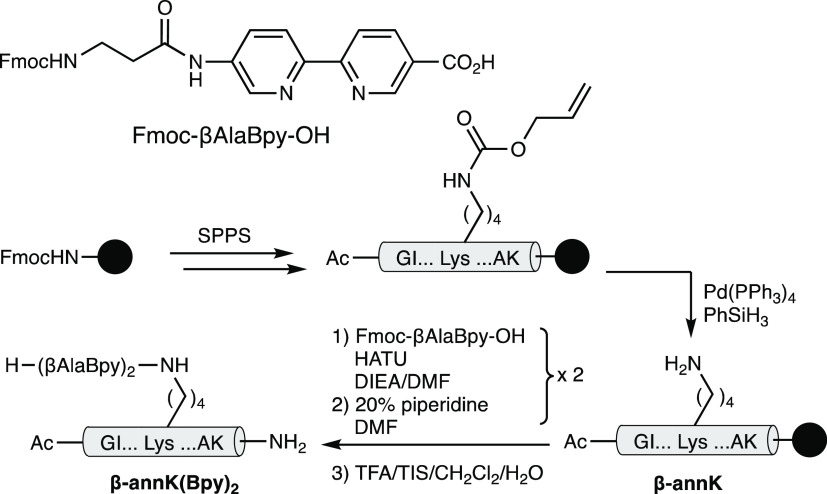
Solid-Phase Peptide Synthesis (SPPS) of the β-Annulus Helicate Precursor Peptide Ligand **β-annK(Bpy)**_**2**_

To obtain the helicate precursor, we first synthesized the peptide Ac-G^48^IS-£^51^-AP-Lys(Alloc)^54^-AQGAK^59^-NH_2_ with the orthogonally protected Lys handle in place of Ser^54^. In addition, the residue Met^51^ was replaced with an isosteric nor-leucine residue (£^51^) to avoid potential oxidation problems.^42^ Finally, for synthetic reasons, and in order to promote the solubility of the helicate precursor peptide, the C-terminal Met^59^ was replaced with an ionizable Lys residue (K^59^).

The target peptide was built following standard solid-phase peptide synthesis protocols,^43^ and once the β-annulus strand was fully assembled, the Alloc group was selectively removed from the Lys side chain under catalytic conditions (Pd(PPh_3_)_4_, PhSiH_3_, Scheme 1), and the metal-chelating 2,2′-bipyridine bulding blocks (Fmoc-βAlaBpy-OH) were sequentially attached to the orthogonally deprotected Lys ^ζ^NH_2_ (Scheme 1). Finally, the deprotection and release of the peptide from the resin was carried out using standard conditions by treatment with an acidic TFA cocktail. The peptide was purified by reverse-phase HPLC, and the identity confirmed by MS (MALDI-TOF).

Having at hand the desired peptide, we next studied the binding of the peptide **β-annK(Bpy)**_**2**_ to Fe(II) and its capacity for templating the formation of the corresponding helicate. For this, we added increasing amounts of (NH_4_)_2_Fe(SO_4_)_2_·6 H_2_O (Mohr’s salt) to a buffered solution of **β-annK(Bpy)**_**2**_ and recorded the progressive decrease in the emission of the Bpy ligand at 402 nm after each addition (Figure 2a). The resulting titration profile could be fitted to a 2:3 interaction model with the *DynaFit* program,^44,45^ with tight dissociation constants, *K*_D1_ = 5.0 ± 3.3 μM and *K*_D2_ = 3.5 ± 0.7 μM for the first and second coordination, respectively. In addition to the spectroscopic data, ESI-MS analysis of the saturated solution confirmed the formation of the expected complex Fe(II)_2_[**β-annK(Bpy)**_**2**_]_3_ with clear peaks corresponding to [M+3H]^3+^ = 1754,5; [M+4H]^4+^ = 1316.2; [M+5]^5+^ = 1053.2 species (see Figure S6, Supporting Information).

**Figure 2:**
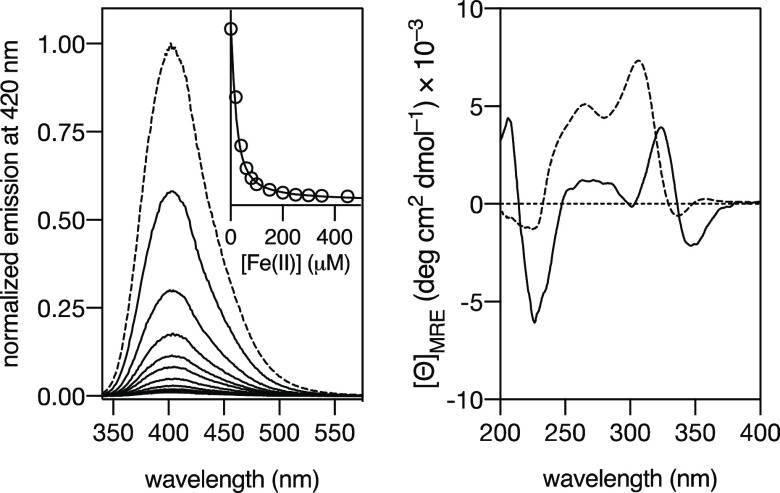
Left: emission spectra of a 20 μM **β-annK(Bpy)**_**2**_ peptide solution (1 mM PBS buffer, 10 mM NaCl, pH 6.5) with increasing concentrations of (NH_4_)_2_Fe(SO_4_)_2_·6H_2_O. Inset: Titration profile of the maximum emission wavelength at 401 nm with increasing concentrations of Fe(II). The best fit according to the 2:3 model in *DynaFit* is also shown. Experimental data points correspond to the average of three independent titrations. Right: CD spectra of a 100 μM **β-annK(Bpy)**_**2**_ peptide solution in HEPES 10 mM buffer, NaCl 100 mM, and pH 6.5 (dashed line), and of the same solution in the presence of 1.5 mM Fe(II) (solid line); the CD intensity of the Fe(II) complex was weaker than that of the isolated peptide so, for clarity purposes, its CD spectrum has been represented multiplied by ten.

Having demonstrated that the **β-annK(Bpy)**_**2**_ peptide self-assembles and coordinates Fe(II) ions, we were interested in evaluating if the chirality of the annulus could be translated into helical chirality in the complex. In other words, if the formation of the helicate Fe(II)_2_[**β-annK(Bpy)**_**2**_]_3_ was stereoselective. Thus, we recorded the circular dichroism (CD) spectra of a 100 μM solution of the **β-annK(Bpy)**_**2**_ peptide and of the same solution in the presence of saturating concentration of Fe(II) (15 equiv). The spectrum of the peptide by itself displayed the typical signature of a β-sheet structure in the far UV region, as well as a clear CD band in the wavelength range of the 2,2′-bipyridine units at ca. 320 nm, which indicated a clear preorganization of these ligands. Furthermore, the CD spectrum in the presence of the Fe(II) showed a more clear Cotton effect in the bipyridine band, which also showed a bathochromic shift; taken together, these changes in the CD spectrum are consistent with the formation of a helicate complex with *P* helicity (ΔΔ*c*hirality in both metal centers).

The stability of the two possible helicate chiralities (*P* and *M*) in Fe(II)_2_[**β-annK(Bpy)**_**2**_]_3_ was assessed by molecular dynamics (MD) simulations in explicit solvent and periodic boundary conditions (see the Supporting Information for computational details). The helical conformation of the helicate unit Fe(II)_2_[(βAlaBpy)_2_]_3_, as well as the octahedral coordination geometry of the Fe(II) ions, was conserved along the simulations, and the trajectories attain relatively stable RMSDs with respect to the initial structures after the first ∼10 ns (2.00 ± 0.52 Å and 1.92 ± 0.46 Å as average for *P* and *M* conformations, respectively); the β-annulus is stabilized after 40 ns with an average RMSD of about 11 Å. Cluster analysis was performed on the full-length MD experiments showing two predominant conformations due to the high mobility of the C-terminal chains that can be directed to the bulk of the solvent or close to the βAlaBpy units, interacting by π-stacking with the Bpy rings and lipophilic contacts with the βAla residue. The conformation in which the C-terminal Lys residues interact with βAlaBpy appear to be more stable for the *P* isomer.

Helicates are known to selectively interact with three-way DNA junctions.^46^ In order to study the binding of the β-annulus helicate to the DNA, we relied on the observation that the Fe(II) complex has a strong quenching effect in nearby fluorophores.^47^ Therefore, we prepared a 2 μM solution of a fluorescein-labeled three-way junction DNA (**twDNA**, FAM-5′-TTTT CAC CGC TCT GGT CCT C-3′; 5′-CAG GCT GTG AGC GGT G-3′; 5′-GAG GAC CAA CAG CCT G-3′) and recorded its emission upon excitation at 490 nm after the addition of successive aliquots of a solution containing the preformed helicate Fe(II)_2_[**β-annK(Bpy)**_**2**_]_3_ (Figure 3, Left). The titration profile of the emission intensity at 515 nm could be fitted to an 1:1 binding mode (**twDNA**/Fe(II)_2_[**β-annK(Bpy)**_**2**_]_3_ complex) with an apparent *K*_D_ of 308 ± 60 nM (Figure 3, Right). In contrast, the titration profile of the helicate with a model duplex DNA could not be fitted to a simple 1:1 binding model and required the introduction of nonspecific interactions in our analysis, in agreement with earlier reports with organic ligand helicates.^48^ Furthermore, the affinity of the helicate for the regular dsDNA was significantly lower than for the three-way junction, with an apparent *K*_D_ of ∼5 μM).

**Figure 3:**
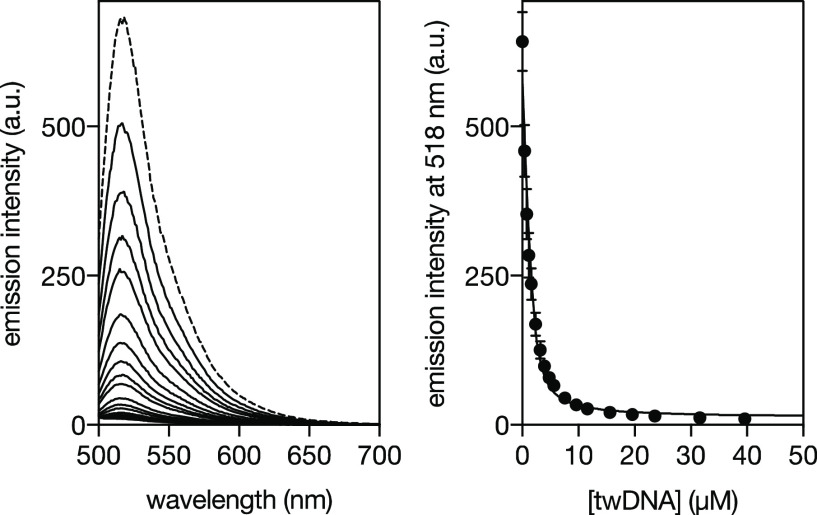
Left: Fluorescence spectra of a 2 μM solution of fluorescein-labeled twDNA in 1 mM PBS buffer, 10 mM NaCl, pH 6.5 (dashed line), and the same solution in the presence of increasing concentrations of the Fe(II)_2_[**β-annK(Bpy)**_**2**_]_3_ helicate (full lines). Right: titration profile (emission intensity at 515 nm) and the best fit to a 1:1 Fe(II)_2_[**β-annK(Bpy)**_**2**_]_3_ helicate/twDNA binding. The experimental points correspond to the mean of three independent titrations.

In addition to the spectroscopic studies, we also analyzed the DNA binding of the helicate Fe(II)_2_[**β-annK(Bpy)**_**2**_]_3_ by electrophoretic mobility assays (EMSA) in polyacrylamide gel under nondenaturing conditions.^49^ In agreement with the fluorescence titration studies, incubation of the target three-way DNA, **twDNA**, with increasing concentrations of the peptide helicate resulted in the appearance of a new slow-migrating band, consistent with the formation of the expected **twDNA**/Fe(II)_2_[**β-annK(Bpy)**_**2**_]_3_ complex (Figure 4, lanes 1–6). Remarkably, no smearing is observed, even at high concentrations of the helicate Fe(II)_2_[**β-annK(Bpy)**_**2**_]_3_, and only one band is formed, thus demonstrating the formation of a unique complex.^50,51^ On the other hand, incubation of a model dsDNA with helicate Fe(II)_2_[**β-annK(Bpy)**_**2**_]_3_ did not induce the formation of new retarded bands, even at high concentrations of the helicate, which clearly confirms the low affinity of this complex for regular B-DNA (Figure 4, lanes 7–10), and supports the model in which the observed fluorescence quenching is due to of low-affinity nonspecific interactions, which are not observed in the more stringent gel electrophoresis conditions.

**Figure 4:**
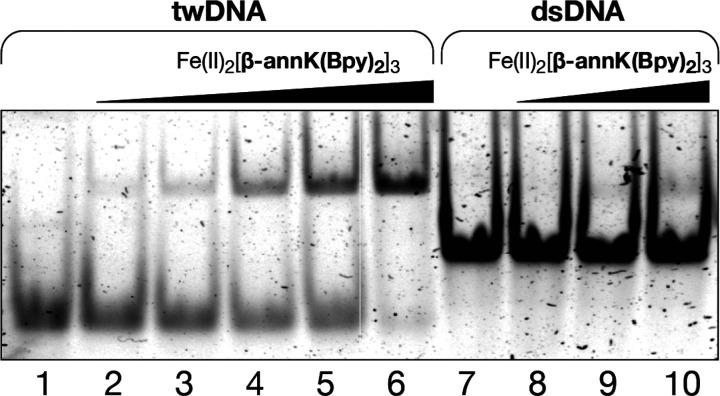
EMSA binding studies results for Fe(II)_2_[**β-annK(Bpy)**_**2**_]_3_ helicate. Lanes 1–6, 200 nM **twDNA** with 0, 150, 250, 500, 1000, and 2000 nM of [**β-annK(Bpy)**_**2**_]_3_ and 20 equiv of (NH_4_)_2_Fe(SO_4_)_2_·6H_2_O in each lane; lanes 7–10, 200 nM **dsDNA** with 0, 500, 1000, and 2000 nM of [**β-annK(Bpy)**_**2**_]_3_ and 20 equiv of (NH_4_)_2_Fe(SO_4_)_2_·6H_2_O in each lane. Samples were resolved on a 10% nondenaturing polyacrylamide gel and 0.5 × TBE buffer over 40 min at 25 °C and stained with SyBrGold (5 μL in 50 mL of 1 × TBE) for 10 min,^52^ followed by fluorescence visualization. Oligonucleotide sequences: **dsDNA** (only one strand), 5′-AAC ACA TGC AGG ACG GCG CTT-3′; **twDNA**, 5′-CAC CGC TCT GGT CCT C-3′; 5′-CAG GCT GTG AGC GGT G-3′; 5′-GAG GAC CAA CAG CCT G-3′.

We have demonstrated that the short β-annulus motif from the Sesbania Mosaic Virus can be modified to direct the stereoselective self-assembly of peptide helicates. Modeling studies support the proposed assembly in which the β-annulus directs the trimerization, the relative orientation of the bipyridine ligands, and induces a *P* helicity in the resulting helicate. Furthermore, the resulting helicate displays high selectivity toward three-way DNA junctions over regular B-dsDNA, as shown by both spectroscopic and electrophoretic assays.

## Supporting Information Available

Detailed experimental procedures for the synthesis of the 2,2′-bipyridine building block, peptide synthesis and characterization, EMSA experiments, and molecular modeling (PDF)
