# Supplementary material for: Stereoselective Self-Assembly of DNA Binding Helicates Directed by the Viral β-Annulus Trimeric Peptide Motif
Source: Bioconjug Chem. 2021 Jul 28;32(8):1564–9. doi: 10.1021/acs.bioconjchem.1c00312 (PMC8485332; doi:10.1021/acs.bioconjchem.1c00312)
Supplement: Supplementary file 1 — bc1c00312_si_001.pdf [file bc1c00312_si_001.pdf]

# Supporting information

## Stereoselective Self-Assembly of DNA Binding Helicates Directed by the Viral β-Annulus Trimeric Peptide Motif

Jacobo Gómez-González,[a,†] David Bouzada,[b,†] Lidia A. Pérez-Márquez,[a,§] Giuseppe Sciortino,[c,‡] Jean-Didier Maréchal,[c] Miguel Vázquez López,[a*] and M. Eugenio Vázquez,[b*]

a. *Centro Singular de Investigación en Química Biolóxica e Materiais Moleculares (CiQUS), Departamento de Química Inorgánica, Universidade de Santiago de Compostela. 15782 Santiago de Compostela, Spain. E-mail: miguel.vazquez.lopez@usc.es*

b. *Centro Singular de Investigación en Química Biolóxica e Materiais Moleculares (CiQUS), Departamento de Química Orgánica, Universidade de Santiago de Compostela. 15782 Santiago de Compostela, Spain.*

c. *Insilichem, Departament de Química, Universitat Autònoma de Barcelona, 08193 Cerdanyola, Spain.*

### Reagents

All the solvents and reagents used in the synthesis of Fmoc-βAlaBpy-OH were obtained from the following commercial sources: *Fisher Chemical* (acetonitrile for HPLC, chloroform, toluene, DMF, methanol, NaOH), *Scharlau* (absolute ethanol), *Sigma Aldrich* (5,5'-dimethyl-2,2'-bipyridine, $KMnO_4$, $SOCl_2$, hydrazine monohydrate, xylene, DIPEA), *Panreac* (celite and $NaNO_2$).

For the synthesis of the peptide ligands all the amino acids—as their Fmoc-protected derivatives—were from *Sigma Aldrich*. The resin employed for the SPPS was H-Rink-Amide *ChemMatrix* 35-100 mesh particle size from *Sigma Aldrich*. For the Alloc deprotection, $Pd(PPh_3)_4$, PhSiH were obtained from *Sigma Aldrich*. Reactives employed in the obtention of the metallopeptides were acquired *Sigma Aldrich* (Mohr's salt).

### Procedures and experimental techniques

#### *Mass spectrometry*

Matrix-Assisted Laser Desorption/Ionization mass spectrometry (MALDI-MS) was performed with a *Bruker Autoflex* MALDI-TOF model in positive scan mode by direct irradiation of the matrix-adsorbed peptide. 4-HCCA (α-cyano-4-hydroxycinnamic acid) was the selected matrix for all these experiments.

#### *UV-Vis Spectroscopy*

UV measurements were made in a *Jasco V-630* spectrophotometer coupled to a *Jasco ETC-717* temperature controller, using a standard *Hellma* semi-micro cuvette (108.002-QS) with a light path of 10 mm. Measurements were made at 20 °C. Acquisition parameters were: 220-700 nm range, scan speed of 200 nm/min, resolution of 0.2 nm.

#### *Fluorescence spectroscopy*

Luminescence experiments were made with a *Varian Cary Eclipse* Fluorescence Spectrophotometer coupled to a *Cary* Single Cell Peltier accessory *(Agilent Technologies)* temperature controller. All measurements were made with a *Hellma* semi-micro cuvette (108F-QS) at 20 °C. The settings for these measurements were adapted depending on the studied system.

#### *Circular Dichroism*

Circular dichroism measurements were made with a *Jasco J-715* coupled to a *Neslab RTE-111* thermostatic water bath, using a *Hellma* 100-QS cuvette (2 mm light pass). Scan speed was 200 nm/min and the obtained spectra are the mean of three accumulations.

### ***HPLC and UHPLC***

*UHPLC-MS & Preparative HPLC*

Peptide analysis was performed by analytical UHPLC-MS with an *Agilent 1200* series LC/MS using a SB $C_{18}$ (1.8 *µ*m, 2.1 × 50mm) analytical column from *Phenomenex*. Standard conditions for analytical UHPLC consisted on a linear gradient from 5% to 95% of solvent B in 12 min at a flow rate of 0.350 mL/min (A: water 0.1% TFA, B: acetonitrile 0.1% TFA). Compounds were detected by UV absorption at 222, 270 and 330 nm. Electrospray Ionization Mass Spectrometry (ESI/MS) was performed with an Agilent 6120 Quadrupole LC/MS model in positive scan mode using direct injection of the purified peptide solution into the MS detector.

Peptide purification was performed by preparative RP-HPLC with a *Waters 1500* series Liquid Chromatograph using a *Sunfire Prep* $C_{18}$ OBD (5 µm, 19 × 150 mm) reverse-phase column from Waters. Standard conditions for preparative RP- HPLC consisted of an isocratic regime during the first 5 min, followed by different linear gradients of solvent B during 30 min (A: water 0.1% TFA, B: acetonitrile 0.1% TFA). The gradients were adjusted for each peptide.

### ***DNA hybridization***

Oligonucleotides were obtained from *Biomers*. Concentration of the oligonucleotides was measured by UV-VIS by using the extinction coefficient given by the supplier.

For the hybridization process, a stochiometric mixture of the DNA strands was prepared in PBS buffer (10 mM, 100 mM NaCl, pH 7.0) and heated at 90 °C for 10 min. The mixture was then slowly cooled down at rt, to obtain the desired tw/ds hybridized DNA.

### ***EMSA experiments***

EMSA were performed with a *BioRad MiniProtean* gel system, powered by an electrophoresis power supplies *PowerPac* Basic model, maximum power 150 V, frequency 50–60 Hz at 140 V (constant V). Binding reactions were performed over 30 min in 1.8 mM Tris-HCl (pH 7.5), 90 mM KCl, 1.8 mM $MgCl_2$, 9% glycerol, 0.11 mg/mL BSA, and 2.2% NP-40. For the experiments we used 200 nM of the DNAs (twDNA and dsDNA), and a total incubation volume of 20 µL. After incubation for 30 min at rt, products were resolved by PAGE using a 10% non-denaturing polyacrylamide gel and 0.5 × TBE buffer (0.445 M Tris, 0.445 M Boric acid) for 35 min at 25 °C, and analyzed by staining with *SybrGold* (*Molecular Probes*: 5 µL in 50 mL of 1 × TBE) for 10 min and visualized by fluorescence (*BioRad GelDoc XR+* molecular imager).

### ***Solid phase peptide synthesis***

*β-annulus synthesis*

C-terminal amide natural T4Ff and β-annulus derivatives were synthesized following standard Fmoc-peptide synthesis protocols on a 0.1 mmol scale using a 0.5 mmol/g loading *H-Rink* amide *ChemMatrix* resin (35–100 mesh size particle) with a *Liberty Lite* automatic microwave assisted peptide synthesizer from CEM Corporation. The amino acids were coupled in 5-fold excess using oxyme as an activating agent. Couplings were conducted for 4 min at 90 °C. Deprotection of the temporal Fmoc protecting group was performed by treating the resin with 20% piperidine in DMF for 1 min at 75 °C. Once the synthesis is finished, the peptide was acetylated with a solution of 0.8 ml AcOH, 2 ml of DIEA/DMF (0.2 M) and 3.2 ml of DMF.

*Deprotection of the Alloc group*

Deprotection of the Alloc group is necessary before the coupling of the two chelating residues of Fmoc-βAlaBpy-OH. Deprotection of the Alloc group was carried out following the methodology proposed by Sainlos *et al.*[1] Thus, a suspension of the resin in DCM was degassed by gently bubbling of $N_2$ for 5 minutes. $Pd(PPh_3)$ (0.8 eq.) and PhSiH (25 eq) were added and the flux of $N_2$ was maintained for 15 min; after this, the resin was washed with DCM three times and the previous process repeated twice.

*Coupling of the Fmoc-βAlaBpy-OH*

Fmoc-βAlaBpy-OH residues were coupled by hand in 4-fold excess using HATU as activating agent. Each amino acid was activated for 1 min in DIEA/DMF 0.2 M (4 mL) before being added onto the resin.

These manual couplings were conducted for 60 min. Deprotection of the temporal Fmoc protecting group was performed by treating the resin with 20% piperidine in DMF for 20 min.

*Cleavage from the resin and final deprotection*

Cleavage and deprotection of the peptide were simultaneously performed using standard conditions by incubating the resin for 2.5 h with an acidic mixture containing 50 µL $CH_2Cl_2$, 25 µL of $H_2O$, 25 µL of TIS (triisopropylsilane), and 900 µL of TFA. The resin was filtered, and the TFA filtrate was concentrated under a nitrogen stream to an approximate volume of 1 mL, and then added onto ice-cold diethyl ether (20 mL). After 10–30 min, the precipitate was centrifuged and washed again with 5 mL of ice-cold ether. The obtained residue was then dissolved in a mixture of $H_2O$ and MeCN and purified by preparative HPLC for the obtention of the pure compounds.

### *Synthesis of Fmoc-βAlaBpy-OH (8)*

The synthesis of this chelating residue was carried out following the methodology recently published by our group with minor modifications.[2] All the obtained products were analyzed by HPLC-MS to test their purity.

*2,2'-bipyridine-5,5'-dicarboxilic acid (**2**)*

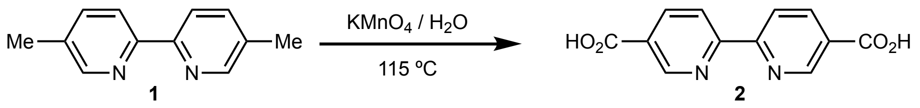


A mixture of 39.0 g of potassium permanganate and 7.0 g of 5,5'-dimethyl-2,2' -bipyridine in 250 mL of $H_2O$ was heated for 2 h (115 °C), cooled at room temperature and filtered trough celite. The filtrate was cooled to 4 °C and acidified with HCl until precipitation of a white solid, which was filtered, washed with water and freeze-dried to afford the desired product in 93% yield (8.7 g)

**$^1$H-NMR** (500 MHz, DMSO-*d*$_6$, δ): 13.50 (br); 9.18 (dd, $^4J$ = 2.15, $^5J$ = 0.8 Hz 2H); 8.55 (dd, $^3J$ = 8.3; $^5J$ = 0.8 Hz, 2H); 8.44 (dd, $^3J$ = 8.3; $^4J$ = 2.15 Hz, 2H).

**$^{13}$C-NMR** (125 MHz, DMSO-*d*$_6$, δ): 165.49, 156.84, 149.82, 137.97, 126.65, 120.62.

**MALDI-TOF** (m/z) $[M+H]^+$ calculated for $[C_{12}H_8N_2O_4]$ 245.0; found 245.0.

*Diethyl [2,2'-bipyridine]-5,5'-dicarboxylate (**3**)*

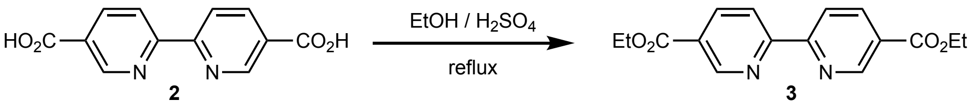


2,2'-bipyridine-5,5'-dicarboxylic acid (10.0 g, 41 mmol) was suspended in 150 mL of absolute ethanol. Concentrated $H_2SO_4$ (20.0 mL) was slowly added and the resulting mixture was refluxed for 18 h. The solution was cooled at rt and added over 400 mL of water at 4 °C causing the precipitation of a white solid, which was filtered, washed with water and lyophilized. 11.5 g (93.0 %)

**$^1$H-NMR** (500 MHz, DMSO-*d*$_6$, δ): 9.20 (dd, $^4J$ = 2.15, $^5J$ = 0.8 Hz 2H); 8.57 (dd, $^3J$ = 8.3; $^5J$ = 0.8 Hz, 2H); 8.46 (dd, $^3J$ = 8.3; $^4J$ = 2.15 Hz, 2H); 4.0 (q, $^3J$ = 7.1 Hz, 4H); 1.37 (t, $^3J$ = 7.1 Hz, 6H).

**$^{13}$C-NMR** (125 MHz, DMSO-*d*$_6$, δ): 164.2, 157.2, 149.8, 138.0, 126.2, 121.0, 61.1, 13.9.

**MALDI-TOF** (m/z) $[M+H]^+$ calculated for $[C_{16}H_{16}N_2O_4]$ 301.1; found 301.1.

*Ethyl 5'-Carbohydrazido-2,2'-bipyridine-5-carboxylate (**4**)*

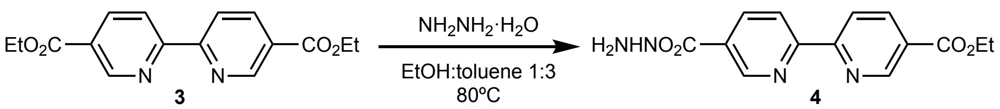


A mixture of diethyl 2,2'-bipyridine-5,5'-dicarboxylate (15.0 g, 50 mmol) and hydrazine hydrate (3.75 mL, 55 mmol) in a solution of EtOH (42 mL) and toluene (128 mL) was heated at 80 °C for 48 h. The

precipitate was filtered, washed with $CHCl_3$ and dried under vacuum 11.4 g (80%). The unreacted diethyl ester was concentrated and mixed again to obtain a global yield of (90%).

**$^1$H-NMR** (500 MHz, DMSO-$d_6$, δ): 1.35 (t, 3H), 4.37 (q, 2H), 8.35 (d, $^3J$ = 8.4 Hz, 1H), 8.45 (dd, $^3J$ = 8.4 Hz, $^4J$) 2 Hz, 1H), 8.52 (d, $^3J$ = 8.4 Hz, 1H), 8.57 (d, $^3J$ = 8.4 Hz, 1H), 9.1 (d, $^4J$ = 2 Hz, 1H), 9.2 (d, $^4J$ = 2 Hz, 1H), 10.1 (br, 1H).

**$^{13}$C-NMR** (125 MHz, DMSO-$d_6$, δ): 14.1, 61.3, 120.9, 120.9, 126.1, 129.6, 136.2, 138.2, 148.1, 150.0, 155.8, 157.8, 163.8, 164.5.

**MALDI-TOF** (m/z) $[M+H]^+$ calculated for $[C_{14}H_{14}N_4O_3]$ 287.1; found 287.1.

*Ethyl 5'-Carbohydrazido-2,2'-bipyridine-5-carboxylate (**5**)*

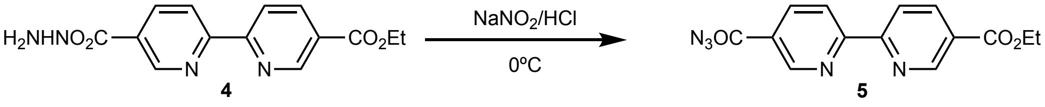


A stirred solution of 5-(ethoxycarbonyl)-5'-carbohydrazido-2,2'-bipyridine (5.7 g, 20 mmol) in concentrated HCl (100 mL) was cooled to 0 °C, and then an aqueous solution of $NaNO_2$ (1.73 g, 25 mmol; 15 mL) was added dropwise, maintaining the temperature at 0 °C. After 60 min, the yellow solution was diluted with water (300 mL) to precipitate the monoester **5** as a white powder, which was filtered, washed with water, and lyophilized 5.5 g (92 %)

**$^1$H-NMR** (500 MHz, Acetone-$d^6$, δ): 9.13 (d, $^4J$ = 1.91 Hz, 1H), 9.10 (d, $^4J$ = 1.91 Hz, 1H), 8.71 (d, $^3J$ = 12.5 Hz, 1H), 8.69 (d, $^3J$ = 12.5 Hz, 1H), 8.517 (m, 2H; 4.31 (q, $^3J$ = 7.15 Hz, 2H); 1.29 (t, $^3J$ = 7.15, 3H).

**$^{13}$C-NMR** (125 MHz, Acetone-$d_6$, δ): 170.8, 164.5, 159.2, 157.7, 150.3, 150.0, 138.0, 137.9, 127.0, 121.3, 61.2, 13.6.

**MALDI-TOF** (m/z) $[M+H]^+$ calculated for $[C_{14}H_{11}N_5O_3]$ 298.1; found 298.1

*Ethyl 5'-(ethoxycarbonyl)amino-2,2'-bipyridine-5-carboxylate (**6**)*

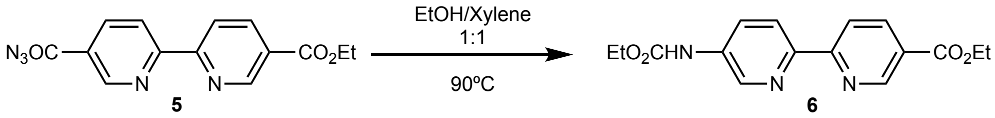


A solution of ethyl 5'-carbazido-2,2'-bipyridine-5-carboxylate (8.6 g, 29 mmol) in a mixture of EtOH (100 mL) and xylene (100 mL) was heated at 90 °C for 4 h. The solvent was evaporated under reduced pressure, and the yellow residue was washed with EtOH and dried in vacuo. 8.21 g (90%).

**$^1$H-NMR** (500 MHz, DMSO-$d_6$, δ): 1.27 (t, $J$ = 7.2 Hz, 3H), 1.35 (t, $J$ = 7.2 Hz, 3H), 4.17 (q, $J$ = 7.2 Hz, 2H), 4.36 (q, $J$ = 7.2Hz, 2H), 8.08 (dd, $^3J$ = 8 Hz, $^4J$ = 2 Hz, 1H), 8.35 (dd, $^3J$ = 8.3 Hz, $^4J$ = 2 Hz, 1H), 8.40 (dd, $^3J$ = 8.3Hz, $^5J$ = 1 Hz, 1H), 8.45 (d, $^3J$ = 8 Hz, 1H), 8.70 (d, $^4J$ = 2 Hz, 1H), 9.12 (d, $^4J$ = 2 Hz, $^5J$ = 1 Hz, 1H), 10.13 (s, 1H).

**$^{13}$C-NMR** (125 MHz, DMSO-$d_6$, δ): 13.6, 13.9, 60.3, 60.6, 119.1, 121.2, 124.46, 124.9, 136.7, 137.4, 139.0, 147.4, 149.4, 153.1, 158.1, 164.2.

**MALDI-TOF** (m/z) $[M+H]^+$ calculated for $[C_{16}H_{17}N_3O_4]$ 316.1; found 316.1.

*5'-Amino-2,2'-bipyridine-5-carboxylic acid hydrochloride (**7**)*

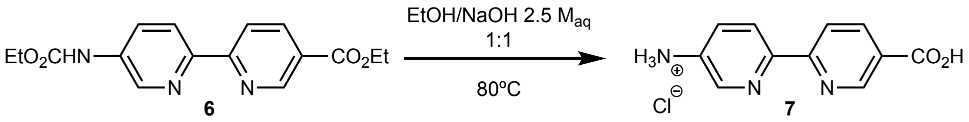


A stirred solution of ethyl 5'-[(ethoxycarbonyl)amino]-2,2'-bipyridine-5-carboxylate (14.55 g, 45.9 mmol) in a mixture of EtOH (50 mL) and 2.5 M aqueous NaOH (50 mL) was heated at 75 °C for 14 h. The

EtOH was concentrated in vacuum, and the aqueous solution was acidified with HCl to afford a bright yellow precipitate, which was filtered, washed with cold water, and lyophilized 9.80 g (85%).

**$^1$H-NMR** (500 MHz, $D_2O$, δ): 7.00 (dd, $^3J$ = 8.5 Hz, $^4J$ = 2.8 Hz, 1H), 7.55, 7.61 (d, $^3J$ = 8.5 Hz, 2H), 7.81 (d, $^4J$ = 2.9 Hz, 1H), 7.98 (dd, $^3J$ = 8.5 Hz, $^4J$ = 2.9 Hz, 1H), 8.65 (d, $^4J$ = 2.8 Hz, 1H).

**$^{13}$C-NMR** (125 MHz, $D_2O$, δ): 123.3d, 126.2, 126.7, 133.8, 139.8, 141.34, 147.5, 147.4, 152.4, 159.6, 176.04.

**MALDI-TOF** (m/z) $[M+H]^+$ calculated for $[C_{11}H_9N_3O_2]$ 216.1; found 216.1.

*Synthesis of Fmoc-βAla5Bpy-OH (8)*

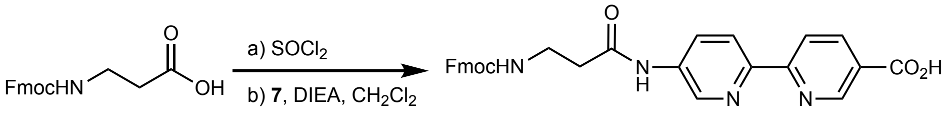


Fmoc-βAla5Bpy-OH, **8**

10 mL of $SOCl_2$ were carefully added at room temperature over 4.58 g (14.72 mmol) of Fmoc-βAla. The solution was stirred for 30 min, the thionyl chloride was evaporated in vacuum and the yellow solid was washed with $CH_2Cl_2$ (3 × 10 mL) and dried under reduced pressure.

The residue was dissolved in 20 mL of $CH_2Cl_2$. Compound (**7**) 3.7 g (14.8 mmol), and 5 mL of DIEA were added, and the resulting suspension was stirred at rt overnight. The solvent was evaporated, the solid was suspended in acetonitrile 0.1 %TFA/$H_2O$ (2:1), centrifuged and washed with acetonitrile and $H_2O$ and lyophilized 6.70 g (89 %).

**$^1$H–RMN** (300 MHz, DMSO-$d_6$, δ): 10.44 (s, 1H); 9.13 (d, $^4J$ = 1.36 Hz, 1H); 8.89 (d, $^4J$ = 2.12 Hz, 1H); 8.42 (m, 3H); 8.25(dd, $^3J$ = 8.48 Hz, $^4J$ = 2.12 Hz, 1H); 7.88 (d, $^3J$ = 7.46 Hz, 2H); 7.67 (d, $^3J$ = 7.38 Hz, 2H); 7.49 (t, $^3J$ = 5.34 Hz, NH); 7.40 (t, $^3J$ = 7.21 Hz, 2H); 7.30 (t, $^3J$ = 7.38 Hz, 2H); 4.30 (d, $^3J$ = 6.95 Hz, 2H); 4.21 (t, $^3J$ = 6.95 Hz, 1H) 3.32 (t, $^3J$ = 6.70 Hz, 2H); 2.60 (t, $^3J$ = 6.70 Hz, 2H).

**$^{13}$C-NMR** (75 MHz, DMSO-$d_6$, δ): 169.76 (C), 168.62 (C), 157.23 (C), 150.94 (C), 149.21 (C), 144.74 (C), 143.47 (C), 142.27 (C), 141.59 (C), 140.28 (CH), 138.29 (CH), 138.08 (CH), 129.73 (CH), 128.09 (CH), 125.96 (CH), 122.14 (CH), 120.87 (CH), 120.79 (CH), 118.73 (CH), 110.34 (CH), 67.38 ($CH_2$), 63.77 ($CH_2$), 47.62 (CH), 41.71 ($CH_2$).

**MALDI-TOF** (m/z) $[M+H]^+$ calculated for $[C_{29}H_{25}N_4O_5]$ 509.1; found 509.1.

### *UHPLC-MS of the purified peptide ligands*

**β-annK(Alloc):** H-GISnLAPK(Alloc)AQGAM-$NH_2$

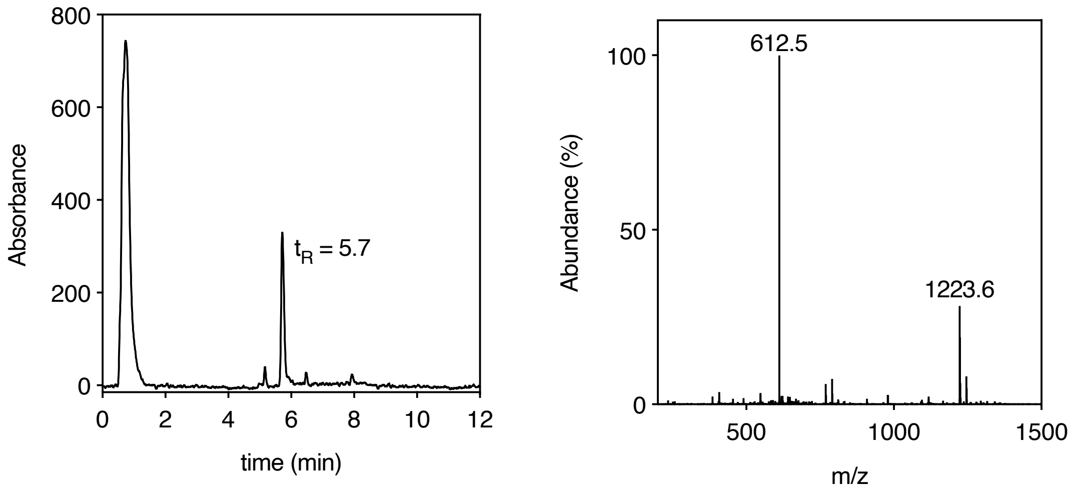


Figure S1. Left: chromatogram of the crude of **β-annK**(Alloc). Right: mass spectra corresponding to the peak wit $t_R$ of 5.7 min.

**HPLC-MS (ESI):** (5-95% B, $t_R$= 5.7 min) Calculated for $C_{54}H_{94}N_{16}O_{16}$ = 1222.70; found $[M+H]^+$ = 1223.6; $[M+2H]^{2+}$ = 612.5.

**β-annK(Bpy)₂:** AcNH-GISnLAPK(βAla5Bpy)$_2$AQGAM-$NH_2$

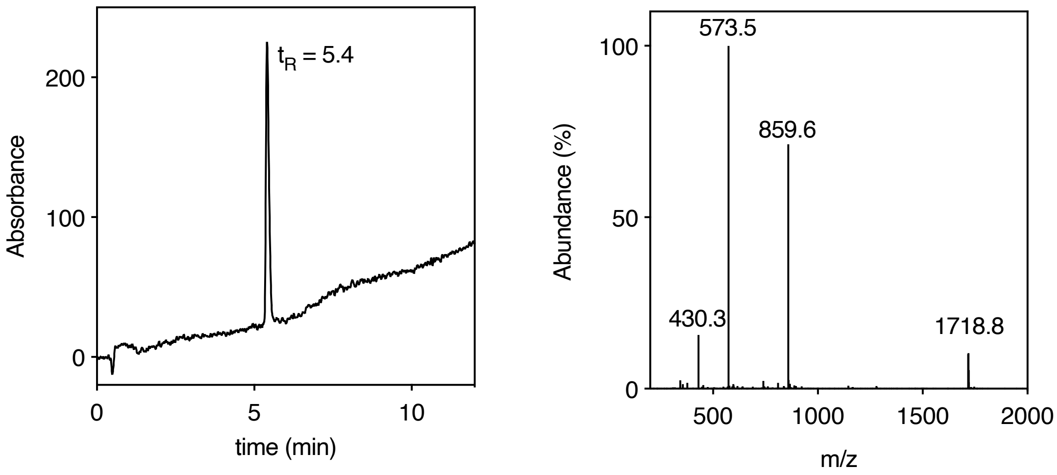


Figure S2. Left: chromatogram of the purified **β-annK(Bpy)₂**. Right: mass spectra corresponding to the peak wit $t_R$ of 5.4 min.

**HPLC-MS (ESI):** (5-95% B, $t_R$= 5.5 min) Calculated for $C_{80}H_{116}N_{24}O_{19}$ = 1717.95; found $[M+H]^+$ = 1718.8; $[M+2H]^{2+}$ = 859.6; $[M+3H]^{3+}$ = 573.5; $[M+4H]^{4+}$ = 430.3.

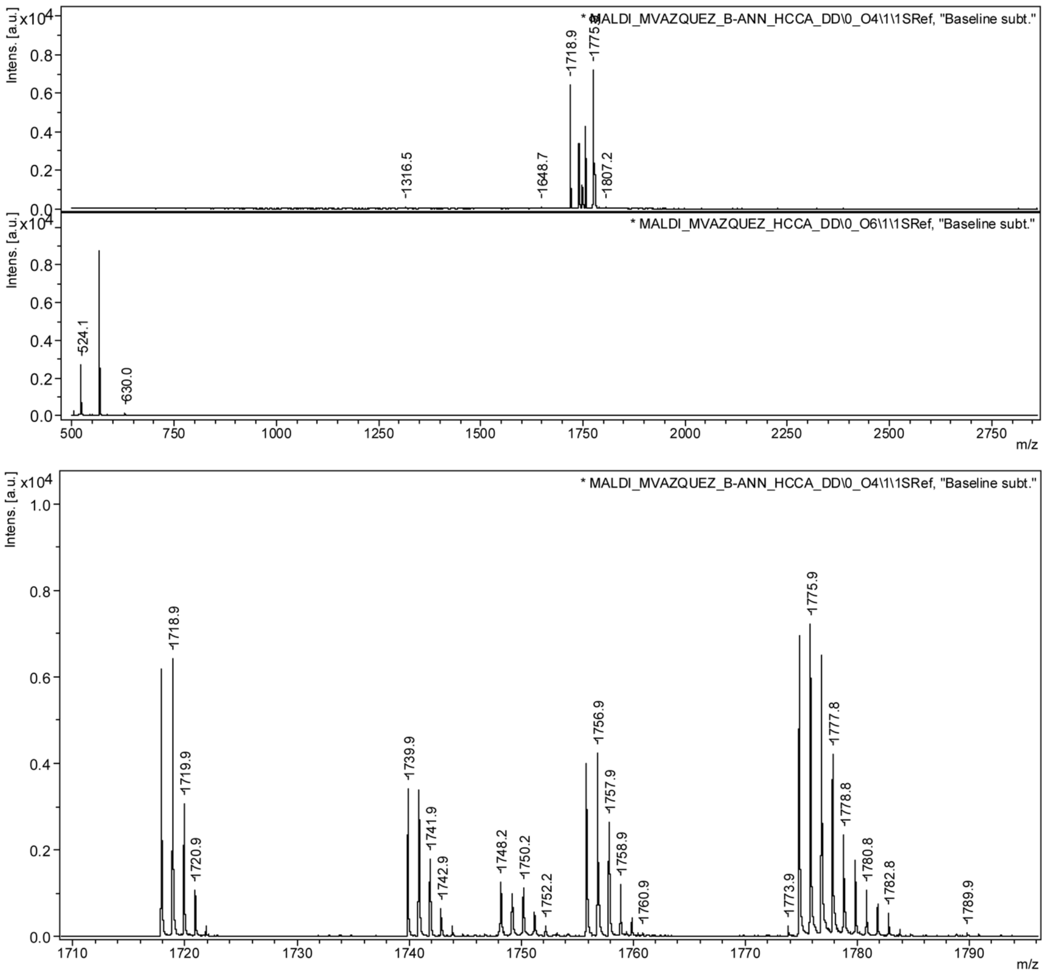


Figure S3. MALDI-TOF of **β-annK(Bpy)₂**. Calculated for $C_{80}H_{116}N_{24}O_{19}$ = 1717.95; found: $[M+H]^+$ = 1717.91; $[M+Na]^+$ = 1739.89; $[M+K]^+$ = 1755.90; $[M+Fe-H]^+$ = 1774.89.

Self-assembly of the **β-annK(Bpy)₂** peptide shows the formation of the trimer as major product, together with higher order assemblies.

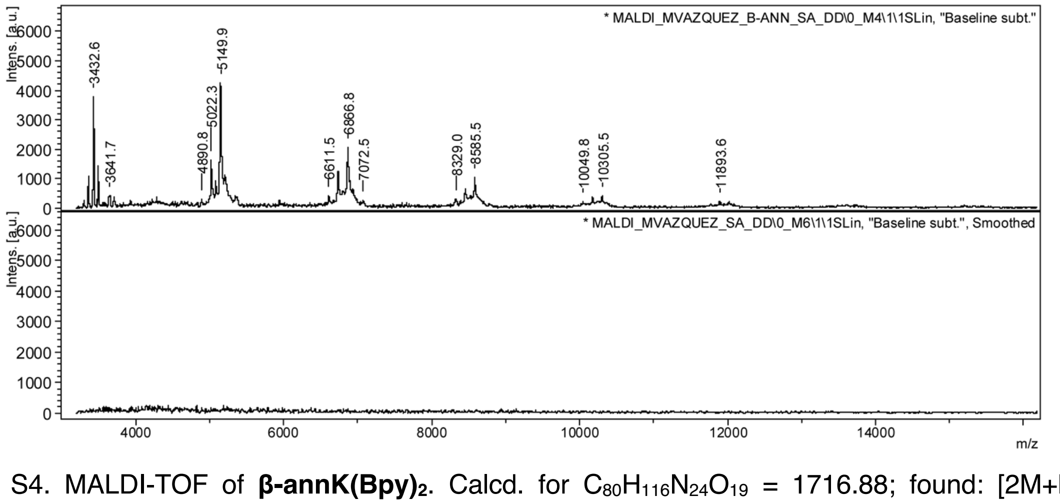


Figure S4. MALDI-TOF of **β-annK(Bpy)₂**. Calcd. for $C_{80}H_{116}N_{24}O_{19}$ = 1716.88; found: $[2M+H]^+$ = 3434.61; $[3M+H]^+$ = 5149.90; $[4M+H]^+$ = 6866.89; $[5M+H]^+$ = 8585.51; $[6M+H]^+$ = 10305.51.

***Synthesis of the Fe(II) peptide helicates***

Onto a 2 μM solution of the peptide ligand in phosphate buffer (1 mM, 10 mM NaCl, pH 6.5), aliquots of a stock solution of $(NH_4)_2Fe(SO_4)_2$ • 6 $H_2O$ (Mohr's salt) was added as source of Fe(II) ions until saturating conditions were reached.

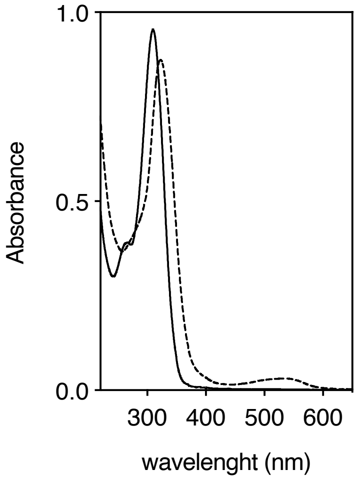


Figure S5. UV-VIS spectra of a 5 μM solution of [**β-annK(Bpy)₂**]₃ ligand before (black solid line) and after the addition of 15 eq equivalents of $(NH_4)_2Fe(SO_4)_2$ · 6 $H_2O$ (black dashed line). Formation of the corresponding Fe(II) metallopeptide was monitored by the appearance of a MLCT band centered at 535 nm.

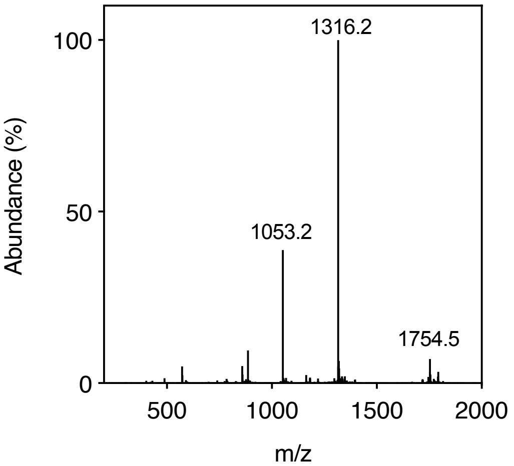


Figure S6. ESI-TOF of $Fe(II)_2$[**β-annK(Bpy)$_2$**]$_3$ obtained upon incubation of a 5 μM solution of [**β-annK(Bpy)$_2$**]$_3$ with 15 eq. of $(NH_4)_2Fe(SO_4)_2$ · 6 $H_2O$. Calcd. For $C_{240}Fe_2H_{348}N_{72}O_{57}$ = 5262,52; found: $[M+3H]^{3+}$ = 1754,5; $[M+4H]^{4+}$ = 1316.2; $[M+5]^{5+}$ = 1053.2.

### *dsDNA/twDNA competition experiment*

Competition experiment showing the preferential binding of the ΛΛ-Fe(II)$_2$[**β-annX(5Bpy)$_2$**]$_3$ helicate to **twDNA** in the presence of **dsDNA**. An equimolar mixture of both DNAs (lane 3) was incubated with the helicate, giving rise to a new band corresponding the ΛΛ-Fe(II)$_2$[**β-annX(5Bpy)$_2$**]$_3$/**twDNA** complex (highlighted) with complete displacement of the free **twDNA**, the band corresponding to the free **dsDNA** is still clearly visible (lane 6).

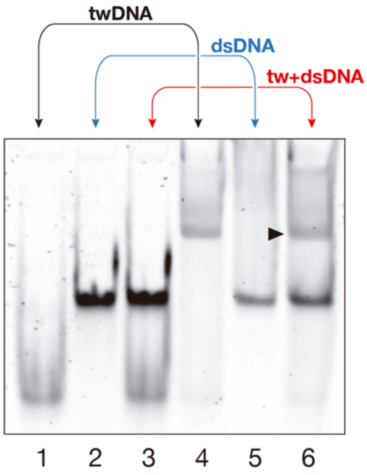


Figure S7. EMSA DNA competition studies results for the ΛΛ-Fe(II)$_2$[**β-annX(5Bpy)$_2$**]$_3$ helicate. Lane 1, 200 nM **twDNA**; lane 2, 200 nM **dsDNA**; lane 3, 200 nM **twDNA** and 200 nM **dsDNA**; lane 4 same as in lane 1 (200 nM twDNA) with 2 $\mu$M of ΛΛ-Fe(II)$_2$[**β-annX(5Bpy)$_2$**]$_3$, lane 5, same as in lane 2 (200 nM **dsDNA**) with 2 $\mu$M of ΛΛ-Fe(II)$_2$[**β-annX(5Bpy)$_2$**]$_3$, lane 6, same as in lane 3 (mixture of 200 nM **dsDNA** and **twDNA**) with 2 $\mu$M of ΛΛ-Fe(II)$_2$[**β-annX(5Bpy)$_2$**]$_3$. Samples were resolved on a 10% nondenaturing polyacrylamide gel and 0.5 x TBE buffer over 40 min at 25 °C and stained with SyBrGold (5 µL in 50 µL of 1 x TBE) foe 10 min, followed by fluorescence visualization. **twDNA**: 5'-CAC CGC TCT GGT CCT C-3'; 5'-CAG GCT GTG AGC GGT G-3'; 5'-GAG GAC CAA CAG CCT G-3'. **dsDNA**: 5'-AAC ACA TGC AGG ACG GCG CTT-3' (only one strand shown).

### *Molecular Modeling*

Molecular Dynamics (MD) simulations were set up with the *xleap*, solvating the structures with a box of pre-equilibrated TIP3P water molecules and the total charge was balanced with chloride ions (*ions94.lib* library). The AMBER99SB force field,[3] was used for the standard residues, while the GAFF force field was adopted for the remaining atoms. Fe-bonding force constants and equilibrium parameters were obtained through the *Seminario* method, using *Gaussian 09*[4] to compute the geometry and harmonic frequencies at DFT level with the B3LYP functional,[5] combined with scalar-relativistic Stuttgart-Dresden SDD pseudopotential and its associated double-ζ basis plus a set of *f* polarization functions for the Fe.[6] The 6-31G(d,p) basis set was used for H, C, O and N. Point charges were derived using the RESP (Restrained *ElectroStatic* Potential) model.[7] The force field building operations were carried out using the MCPB.py.[8] The solvent and the whole system were sequentially submitted to 3000 energy minimization steps to relax possible steric clashes. Then, thermalization of water molecules and side chains was achieved by increasing the temperature from 100 K up to 300 K. MD simulations under periodic boundary conditions were carried out during 100 ns with OpenMM engine,[9] through OMMProtocol.[10] Analysis of the trajectories was carried out by means of *cpptraj* implemented in ambertools16.

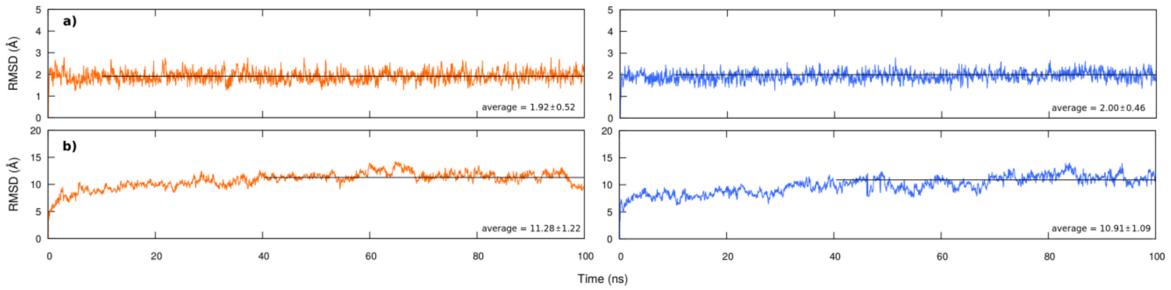


Figure S8: Computed RMSD along the MDs trajectories for ΔΔ- (in orange) and ΛΛ- (in blue) enantiomers of Fe(II)$_2$[**β-annK(Bpy)**$_2$]$_3$ using the minimized initial structures as a reference: a) RMSD relative to the helicate region, and b) to the whole system. The dispersion in terms of 2σ is also reported.

### References


1 M. Sainlos, B. Imperiali, *Nat. Protoc.* **2007**, *2*, 3201–3209.

2 I. Gamba, G. Rama, E. Ortega-Carrasco, J.-D. Maréchal, J. Martínez-Costas, M. E. Vázquez, M. Vázquez López, *Chem. Commun.* **2014**, *50*, 11097-11100

3 Viktor, H.; Robert, A.; Asim, O.; Bentley, S.; Adrian, R.; Carlos, S., Comparison of multiple amber force fields and development of improved protein backbone parameters. *Proteins: Struct., Funct., Bioinf.* **2006,** *65*, 712-725.

4 Frisch, M. J.; Trucks, G. W.; Schlegel, H. B.; Scuseria, G. E.; Robb, M. A.; Cheeseman, J. R.; Scalmani, G.; Barone, V.; Mennucci, B.; Petersson, G. A.; Nakatsuji, H.; Caricato, M.; Li, X.; Hratchian, H. P.; Izmaylov, A. F.; Bloino, J.; Zheng, G.; Sonnenberg, J. L.; Hada, M.; Ehara, M.; Toyota, K.; Fukuda, R.; Hasegawa, J.; Ishida, M.; Nakajima, T.; Honda, Y.; Kitao, O.; Nakai, H.; Vreven, T.; Montgomery, J. A., Jr.; Peralta, J. E.; Ogliaro, F.; Bearpark, M.; Heyd, J. J.; Brothers, E.; Kudin, K. N.; Staroverov, V. N.; Keith, T.; Kobayashi, R.; Normand, J.; Raghavachari, K.; Rendell, A.; Burant, J. C.; Iyengar, S. S.; Tomasi, J.; Cossi, M.; Rega, N.; Millam, J. M.; Klene, M.; Knox, J. E.; Cross, J. B.; Bakken, V.; Adamo, C.; Jaramillo, J.; Gomperts, R.; Stratmann, R. E.; Yazyev, O.; Austin, A. J.; Cammi, R.; Pomelli, C.; Ochterski, J. W.; Martin, R. L.; Morokuma, K.; Zakrzewski, V. G.; Voth, G. A.; Salvador, P.; Dannenberg, J. J.; Dapprich, S.; Daniels, A. D.; Farkas, Ö.; Foresman, J. B.; Ortiz, J. V.; Cioslowski, J.; Fox, D. J., *Gaussian 09, revision c.01*. Gaussian, Inc.: Wallingford, CT, 2010

5 Yanai, T.; Tew, D. P.; Handy, N. C., A new hybrid exchange–correlation functional using the coulomb-attenuating method (cam-b3lyp). *Chem. Phys. Lett.* **2004,** *393*, 51-57.

6 Ehlers, A.; Böhme, M.; Dapprich, S.; Gobbi, A.; Höllwarth, A.; Jonas, V.; Köhler, K.; Stegmann, R.; Veldkamp, A.; Frenking, G., A set of f-polarization functions for pseudo-potential basis sets of the transition metals sc-cu, y-ag and la-au. *Chem. Phys. Lett.* **1993,** *208*, 111-114.

7 Bayly, C. I.; Cieplak, P.; Cornell, W.; Kollman, P. A., A well-behaved electrostatic potential based method using charge restraints for deriving atomic charges: The resp model. *J. Phys. Chem.* **1993,** *97*, 10269-10280.

8 Li, P.; Merz Jr, K. M., Mcpb. Py: A python-based metal center parameter builder. ACS Publications: 2016.

9 Eastman, P.; Swails, J.; Chodera, J. D.; McGibbon, R. T.; Zhao, Y.; Beauchamp, K. A.; Wang, L.-P.; Simmonett, A. C.; Harrigan, M. P.; Stern, C. D.; Wiewiora, R. P.; Brooks, B. R.; Pande, V. S., Openmm 7: Rapid development of high-performance algorithms for molecular dynamics. *PLoS Comput. Biol.* **2017,** *13*, e1005659.

10 D.A. Case, R. M. B., W. Botello-Smith, D.S. Cerutti, T.E. Cheatham, III, T.A. Darden, R.E. Duke, T.J. Giese, H. Gohlke, A.W. Goetz, N. Homeyer, S. Izadi, P. Janowski, J. Kaus, A. Kovalenko, T.S. Lee, S. LeGrand, P. Li, C. Lin, T. Luchko, R. Luo, B. Madej, D. Mermelstein, K.M. Merz, G. Monard, H. Nguyen, H.T. Nguyen, I. Omelyan, A. Onufriev, D.R. Roe, A. Roitberg, C. Sagui, C.L. Simmerling, J. Swails, R.C. Walker, J. Wang, R.M. Wolf, X. Wu, L. Xiao, D.M. York and P.A. Kollman *Amber 16*, University of California, San Francisco, 2016.
